# Sex and Age Differences in Ketamine Efficacy and Safety in Chronic Pain Alleviation

**DOI:** 10.3390/jcm14124269

**Published:** 2025-06-16

**Authors:** Gisèle Pickering, Marion Voute, Marc Sorel, Bruno Pereira, Thibault Riant

**Affiliations:** 1 University Hospital Clermont-Ferrand, Inserm CIC 1405, Platform of Clinical Investigation, Platform of Clinical Investigation, F-63000 Clermont-Ferrand, France; gisele.pickering@uca.fr; 2 Clinical Research and Innovation Department, University Hospital Clermont-Ferrand, F-63000 Clermont-Ferrand, France; bpereira@chu-clermontferrand.fr; 3 Centre de la Douleur, Centre Hospitalier Nemours, 77140 Nemours, France; m.sorel@ch-sud77.fr; 4 Unité Douleur, Le Confluent, Catherine de Sienne Center, 44000 Nantes, France; thibault.riant@groupeconfluent.fr

**Keywords:** refractory pain, sex, age, ketamine, depression

## Abstract

**Background:** Ketamine use for chronic pain and depression has increased worldwide, but sex differences in its efficacy and safety have been little studied; this study examines existing evidence to address this gap. **Methods:** A prospective, multicenter, one-year observational study in 585 chronic pain patients was performed; 256 patients had one administration of ketamine and 329 had two or more. The primary outcome looked at was mean pain intensity (0–10), assessed every month for 1 year by telephone. Secondary outcomes included measures of depression and anxiety (assessed using the Hospital Anxiety and Depression Scale), quality of life (evaluated with the 12-item Short Form Health Survey), total ketamine dosage, reported adverse effects, and concomitant treatments. Platform of Clinical Investigation, No sex or age differences were observed in ketamine efficacy in terms of pain (mean variation in women vs. men; effect size −0.5 (95% confidence interval −0.6 to −0.4) vs. −0.5 (95% confidence interval −0.7 to −0.3), *p* = 0.248) or the evolution of anxiety (*p* = 0.135) among the 585 patients. Women reported more adverse events than men (19% vs. 13%, *p* = 0.002). In the subgroup of 329 patients, no differences were observed in any variables, but a single ketamine administration may be more useful in men than in women (*p* = 0.032), especially in younger men (*p* = 0.045). **Conclusions:** Repeated ketamine administration displayed no sex or age differences in efficacy in the treatment of pain, anxiety or depression.

## 1. Introduction

Chronic pain impacts more than 20% of the global population and is closely linked to substantial reductions in quality of life, as well as an increased prevalence of mental health issues, such as depression and anxiety. Treatment failure or a poor response to recommended treatments (antidepressants, antiepileptics and opioids) is frequent and affects more than 60% of patients. In these situations, other options, such as ketamine, an anesthetic and non-selective N-methyl-D-aspartate receptor (NMDAR) antagonist [1,2,3], may be offered for chronic refractory pain, although it is an off-label use. Ketamine reduces excitatory synaptic transmission and postsynaptic calcium signaling and is used for neuropathic chronic pain or complex regional pain syndrome [4], with a rising potential in the treatment of suicidal ideation and refractory depression. The authors of recent works [1,3] have stressed specificities for ketamine efficacy in patients with chronic pain. Distinct trajectories of pain and predictive markers of ketamine response have been described in previous studies [1], and depression (rather than the ketamine dose or anxiety) has been identified as a mediating factor in the relationship between ketamine administration and pain reduction [3]. Indeed, depression is a frequent consequence of chronic pain [5], with a bidirectional interaction [3], and ketamine has demonstrated promising effects as a rapid-acting antidepressant when administered as a therapeutic strategy for managing mood disorders [6].

Sex/gender is defined as a combination of biological characteristics linked to physical and physiological traits [7]. For decades, the role of sex/gender in various outcomes, such as pain perception, chronic pain prevalence, medication use and response to treatment, and adverse drug events [8,9,10,11], has been evident. In view of the differences in behavior and clinical conditions between men and women, which are partly explained by biological parameters, brain molecular organization and the chronic pain phenotype, the impact of sex/gender is very important to study [12,13,14,15]. Other parameters, such as socioeconomic status and psychological factors, may also explain these differences [8,16].

Strong female biases have been consistently observed in chronic pain situations such as fibromyalgia and endometriosis [17], as well as in mood disorders and depressive symptoms [18]. However, the findings from pain studies are rarely disaggregated by sex. Sex and gender differences in analgesia, including with antidepressants [19], have also been described, but sex-specific responses to ketamine in the management of chronic refractory pain have largely been understudied. Studies on sex differences in ketamine analgesia in animals [20,21,22,23] have not been carried out in chronic pain situations, but rather in antinociception conditions. In humans, studies have been conducted on healthy volunteers or in acute situations [24,25,26]. A recent systematic review of mood disorders reported no sex differences in the antidepressant response to ketamine, but a more frequent response was observed in males at 7 days post-infusion [6].

The analgesic effects of ketamine may be modulated by age due to both age-related physiological alterations and pharmacokinetic and pharmacodynamic changes [27,28]. Aging is associated with changes in liver function, blood flow and the activity of cytochrome P450 (CYP) enzymes, particularly CYP2B6 and CYP3A4, which are expressed and catalyze reactions with a high degree of variability in humans [29,30]. Moreover, hepatic metabolism and renal clearance of ketamine tend to decline with age, potentially affecting drug exposure and clinical response [31]. Despite these considerations, age-related differences in ketamine analgesia remain underexplored in the context of chronic pain. A few studies have hinted at reduced responsiveness in older adults, possibly due to neurodegenerative changes or altered receptor sensitivity, but the evidence is still sparse and inconclusive [32,33]. More targeted research is warranted to clarify how aging may influence ketamine’s analgesic properties in chronic pain populations. However, age differences in analgesia with ketamine treatment for chronic pain have been little studied.

The present one-year prospective study involving 585 patients with chronic refractory pain was conducted to identify, for the first time, sex- and age-related differences in ketamine efficacy and safety. Other pain and emotional parameters were also analyzed according to sex and age.

## 2. Materials and Methods

### 2.1. Study Design

This observational, prospective, multicenter study was carried out in thirty pain clinics in France. The participating centers were mainly hospital pain clinics with dedicated clinical research teams, including on-site clinical research associates. Patients were included in the course of their standard care with no added interventions, according to the schedule of the medical practitioners. Patient follow-up was conducted via telephone over a one-year period by the Clinical Investigation Center Inserm 1405 at Clermont-Ferrand University Hospital. The study received ethical approval from the relevant committees (CCTIRS, CNIL and CPP Sud-Est, France) and was registered at ClinicalTrials.gov (NCT03319238).

### 2.2. Study Population

Patients eligible for inclusion were men and women aged 18 years or older who had been experiencing chronic pain for more than six months. This included individuals with peripheral or central neuropathic pain, fibromyalgia, complex regional pain syndrome or other chronic pain conditions. Eligibility further required that ketamine was indicated as part of their therapeutic management. Patients were recruited at the pain clinics where they usually received treatment. The clinician evaluated the eligibility criteria, explained the objectives of the study and provided the patient with information and a non-opposition form. The investigator specified to the patient that they could refuse to participate in the study.

The study was carried out on 585 patients: data for 256 patients who received only one ketamine administration [1] and 329 patients with repeated ketamine administration and in-patient care [3] have been published, but to date, sex and age-related factors have not been analyzed.

### 2.3. Study Drug and Its Administration

Only (R,S)-ketamine was available in France at the time of this study. The pain clinics implemented their own (R,S)-ketamine treatment protocols, which differed in dosage, treatment duration, frequency and administration method (for example, a single 0.2 mg/kg dose over 40 min, or 0.1 mg/kg administered once weekly for eight weeks, either intravenously or subcutaneously). The total cumulative dose was calculated in milligrams: for instance, an individual person weighing 70 kg receiving 0.5 mg/kg once per month over three months corresponds to a cumulative dose of 105 mg.

### 2.4. Follow-Up Procedure

At inclusion and prior to ketamine administration, a standardized form was completed to gather baseline information, including demographics, ketamine exposure status, administration method, pain characteristics, other analgesic treatments and questionnaire responses. An initial follow-up phone call was conducted one week after ketamine administration. Subsequently, patients were monitored for one year through monthly telephone interviews to collect longitudinal data on pain intensity, questionnaire responses, other pharmacological and non-pharmacological treatments and any adverse events.

### 2.5. Study Objectives and Endpoints

The primary aim of this study was to investigate whether and how sex and age influence pain relief following ketamine treatment over a one-year period. The main outcome measured was the average pain intensity during this time, using the numerical pain rating scale ranging from 0 (no pain) to 10 (worst pain imaginable).

The secondary aims were to evaluate the effects of sex and age on changes in depressive and anxiety symptoms, as well as quality of life, for a follow-up period of one year of ketamine treatment. Depression and anxiety were assessed using the Hospital Anxiety and Depression Scale (HADS), with scores from 0 to 21 (≤7 indicating no pathology; 8–10 suggesting possible cases; ≥11 indicating probable cases). Quality of life was evaluated via the 12-item Short Form Health Survey (SF-12), which provides mental and physical component scores. Adverse events and concomitant drug and nondrug treatments are not described in this ancillary study, but these results can be found in the initial study [1].

### 2.6. Sample Size

This study involved an ancillary analysis based on data from two previously published works by our team. As the sample was predetermined, no formal sample size calculation was required for inclusion. However, before conducting the present analysis, we performed a prospective power evaluation based on the characteristics of the available dataset. According to Cohen’s effect size recommendations [30] (small: d = 0.2; medium: d = 0.5; large: d = 0.8), and assuming a standard deviation of 2, a two-sided type I error of 0.001 (adjusted for multiple comparisons) and an intra-individual correlation of 0.5, the available sample provided sufficient power (>90%) to detect medium-to-large differences (effect-size greater than 0.5) in pain, anxiety and depression outcomes across sex (76% women) and age groups (53% ≥ 50 years old).

### 2.7. Statistical Analysis

Continuous data are expressed as the mean ± standard deviation according to the statistical distribution. The assumption of normality was assessed by using the Shapiro–Wilk test.

The primary objective of this ancillary analysis was to identify for the first time sex- and age-related differences in ketamine efficacy and safety, i.e., to investigate the influence of sex, age and their interaction (sex × age) on the response to ketamine treatment in terms of pain, anxiety and depression. Variables such as the number of ketamine administrations, route (intravenous vs. subcutaneous) and dosing regimens, were considered secondary and exploratory factors. These were not the focus of the primary hypothesis, but they were accounted for as rigorously as possible using multivariable models to adjust for potential confounding.

The continuous variables were compared between independent groups (cohorts in terms of sex, age < or ≥ 50 years old according to clinical relevance and statistical distribution) by using Student’s *t*-test or, if the assumptions of the t-test were not met, the Mann–Whitney test. Homoscedasticity was analyzed using the Fisher–Snedecor test. The comparisons between the aforementioned groups were carried out using chi-squared or Fisher’s exact tests for categorical variables.

To analyze longitudinal data (i.e., pain, depression and anxiety evolution), random-effects models for repeated data were generated, with time as a fixed effect and patient as a random effect, to account for between- and within-patient variability. More precisely, changes between groups (sex and age) were compared using the following fixed effects: group, time-point evaluation and their interaction. Age x sex interaction was also analyzed. A Sidak’s type I error correction was applied to perform multiple comparisons. The normality of residuals was studied using the Shapiro–Wilk test. When appropriate, a logarithmic transformation was applied to achieve the normality of the dependent outcome. The results were expressed as effect sizes and 95% confidence intervals and were interpreted according to Cohen’s recommendations [34].

As women and men were not comparable in terms of pathology (fibromyalgia vs. neuropathic pain), a sensitivity analysis was conducted using a propensity score computed to limit these differences matching women and men according to pathology and age. More precisely, nearest neighbor pair matching without replacement within specified calipers of the propensity score was applied. Therefore, the matchit command from the MatchIt package (R software 4.2.2) was used to perform this analysis with a caliper width of 0.2. An approach based on bootstrap estimates, a well-known resampling method for estimating the standard error of estimated statistics and for constructing confidence intervals, was then performed.

As the random effects were robust in the presence of missing data, all analyses described above were conducted on available data. However, sensitivity analyses were carried out to evaluate the statistical nature of missing data and their possible impact on the results. According to these analyses, the missing data were considered differently depending on whether they were during the follow-up or for patients who were lost to follow-up. Therefore, only missing data during the follow-up were imputed using the LOCF procedure, and missing data after the last contact were not replaced. The findings from these analyses were not altered.

Statistical analyses were performed using Stata software (version 15, StataCorp, College Station, TX, USA) and R software (https://cran.r-project.org/, accessed on 27 March 2025) All tests were two-sided, with a type I error set at 5%, and a correction applied for multiple comparisons, when necessary.

## 3. Results

### 3.1. Patient Characteristics

**Figure 1:**
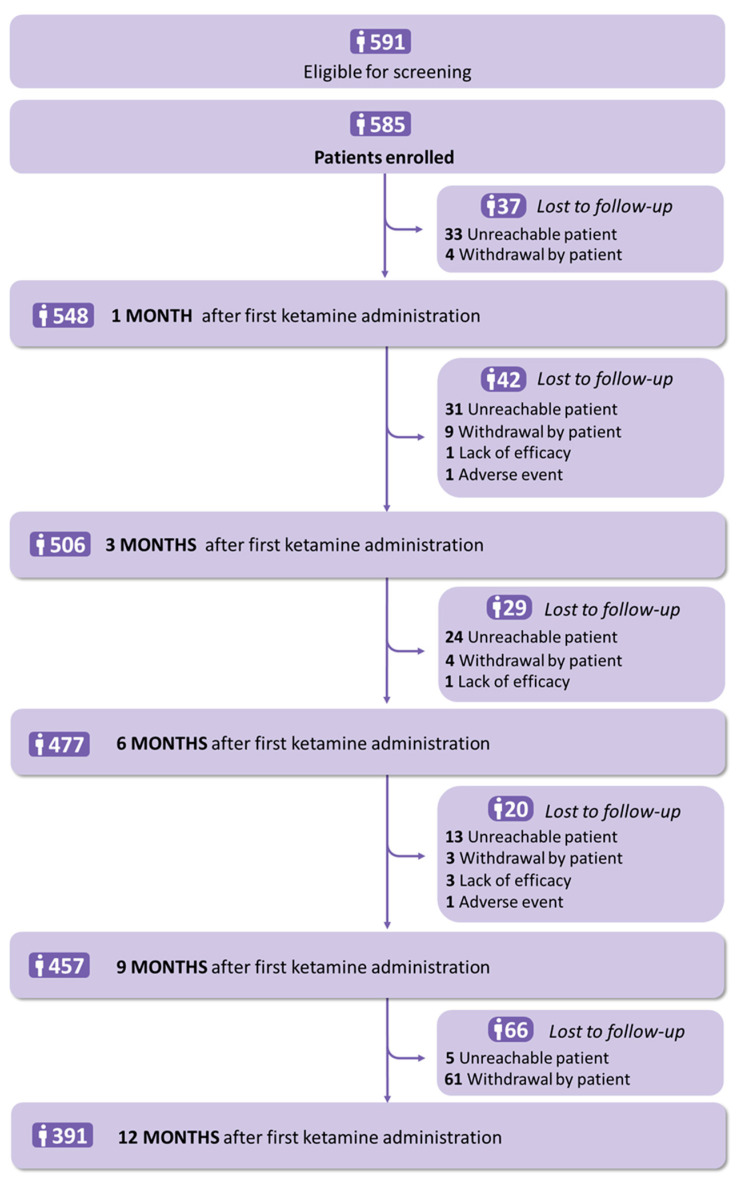
Flowchart.

From 7 July 2016 to 21 September 2017, a total of 585 patients were enrolled, with a mean (SD) age of 51.1 (11.2) years; among them, 443 were women (75.7%) and 142 were men (24.3%). All participants received at least one dose of ketamine. Details of the participants’ characteristics are shown in Table 1 and Figure 1. At baseline, 251/443 women (56.7%) presented with fibromyalgia and 66/142 men (46.5%) with peripheral neuropathic pain (*p* < 0.001). The mean NPRS score was higher in women than in men (mean (SD) pain intensity; 6.9 (1.8) vs. 6.4 (1.7), respectively; *p* < 0.01). The mean HADS anxiety score was higher in women than in men (mean (SD) anxiety score. 10.8 (4.3) vs. 8.9 (4.3), respectively; *p* < 0.001), with definitive cases (score ≥ 11) in 219/416 women (52.6%) and 49/138 men (*p* < 0.001). Concerning concomitant treatment, 31/127 men (22.6%) and 56/437 women (12.8%) reported using step 3 opioids (*p* < 0.01); 296/437 women (67.7%) and 77/137 men reported using antidepressants (*p* < 0.05). All other parameters were not significantly different between the sexes.

### 3.2. Sex and Age Influences in the Total Sample (n = 585)

**Figure 2:**
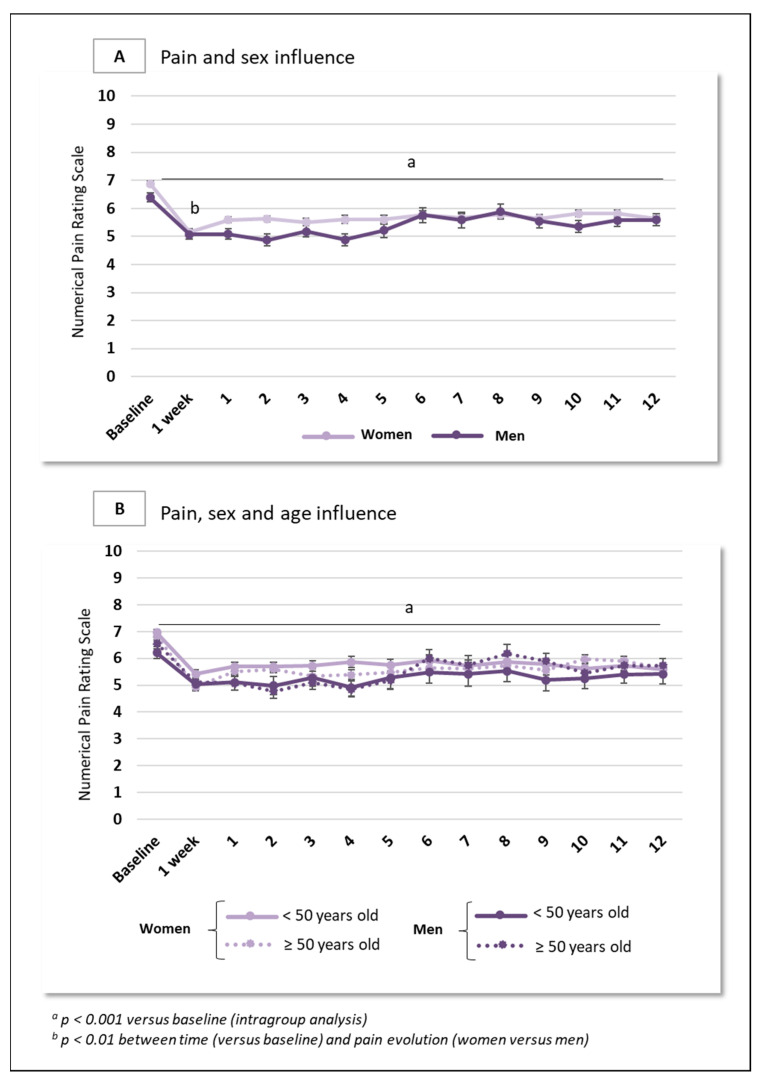
Changes in pain scores according to sex and age in 585 patients with chronic refractory pain. Data are presented as mean (SEM). (**A**). Pain evolution over one year depending on sex. The light line represents women (*n* = 422) and dark line represents men (*n* = 133). (**B**). Pain evolution over one year depending on sex and age. The light full line represents women < 50 years old (*n* = 197); the light dotted line represents women ≥ 50 years old (*n* = 225). The dark full line represents men < 50 years old (*n* = 63); the dark dotted line represents men ≥ 50 years old (*n* = 70).

Between baseline and month 12, the average NPRS score among women diminished from 6.9 (1.8) to 5.6 (2.1), with a mean variation of −1.3 (effect size −0.5 (95% confidence interval −0.6 to −0.4), *p* < 0.001) while pain intensity in men decreased from 6.4 (1.7) to 5.6 (2.1), with a mean variation of −0.8 (effect size −0.5 (95% confidence interval −0.7 to −0.3), *p* < 0.001). The evolution of pain over one year did not differ significantly between women and men (Figure 2A). When pain was considered in two groups (<50 years; ≥50 years), the mean NPRS score in women aged < 50 years decreased from 7.0 (1.7) to 5.6 (2.0), with a mean variation of −1.4 (effect size −0.6 (95% confidence interval −0.8 to −0.5), *p* < 0.001), while pain intensity in women aged ≥ 50 years decreased from 6.8 (1.8) to 5.7 (2.1), with a mean variation of −1.1 (effect size −0.5 (95% confidence interval −0.6 to −0.3), *p* < 0.001). Among men aged < 50 years, pain intensity decreased from 6.2 (1.7) to 5.4 (2.4), with a mean variation of −0.8 (effect size −0.5 (95% confidence interval −0.8 to −0.2), *p* < 0.001). Pain intensity in men aged ≥ 50 years similarly decreased from 6.6 (1.7) to 5.7 (2.0), with a mean variation of −0.9 (effect size −0.5 (95% confidence interval −0.7 to −0.2), *p* < 0.001) (Figure 2B).

Between baseline and month 12, the mean HADS depression score in women decreased from 9.1 (4.4) to 7.5 (4.8), with a mean variation of −1.6 (effect size −0.4 (95% confidence interval −0.5 to −0.3), *p* < 0.001), while the depression score in men decreased from 8.7 (4.2) to 6.5 (4.8), with a mean variation of −2.2 (effect size −0.7 (95% confidence interval −0.9 to −0.5), *p* < 0.001). The evolution of depression over one year differed significantly between women and men (*p* = 0.006) (see Supplementary Figure S1A).

Between baseline and month 12, the mean HADS anxiety score in women decreased from 10.8 (4.2) to 9.0 (4.6), with a mean variation of −1.8 (effect size −0.6 (95% confidence interval −0.7 to 0.5), *p* < 0.001), while the anxiety score in men decreased from 8.9 (4.3) to 6.9 (4.3), with a mean variation of −2.0 (effect size −0.6 (95% confidence interval −0.8 to −0.4), *p* < 0.001). The evolution of anxiety over one year did not differ significantly between women and men (see Supplementary Figure S1B).

Concerning safety and the follow-up of adverse events over one year, 19% of women and 13% of men reported one adverse event (*p* = 0.002, odds ratio = 0.57). Women more frequently reported headaches, fatigue, urinary infection, sweating and fever as adverse events compared to men (8.6% vs. 5.3%, *p* = 0.005).

The cumulative ketamine dose was not different between sexes in the total sample.

### 3.3. Sex and Age Influences in the Cohort Given to Repeated Ketamine Administration (n = 329)

Patients in the cohort with repeated ketamine administration received at least two ketamine administrations over one year. As in the total sample, the evolution of pain over one year did not differ significantly between women and men (see Supplementary Figure S2A), nor with age (see Supplementary Figure S2B). As shown in Supplementary Figure S3, HADS depression and anxiety scores did not differ depending on sex over one year. The cumulative ketamine dose, number of administrations, naïve ketamine profile and route of administration were not different between sexes.

### 3.4. Sex and Age Influences in the Cohort with a Single Ketamine Administration (n = 256)

The cohort with single ketamine administration corresponded to patients who received only one ketamine administration at baseline. Details of the participants’ characteristics are shown in Supplementary Table S1. There were sex differences in the pain type: 101/194 (52.1%) women had fibromyalgia as the main pain type while 13/62 (21.0%) men had peripheral neuropathic pain (*p* < 0.001). The mean HADS anxiety score was higher in women than in men (mean (SD) anxiety score: 10.6 (4.3) vs. 9.1 (4.7), respectively; *p* = 0.020), with 87/180 (48.3%) women and 24/61 men in definite cases (score ≥ 11) (*p* = 0.023). All other parameters were not significantly different between the sexes.

**Figure 3:**
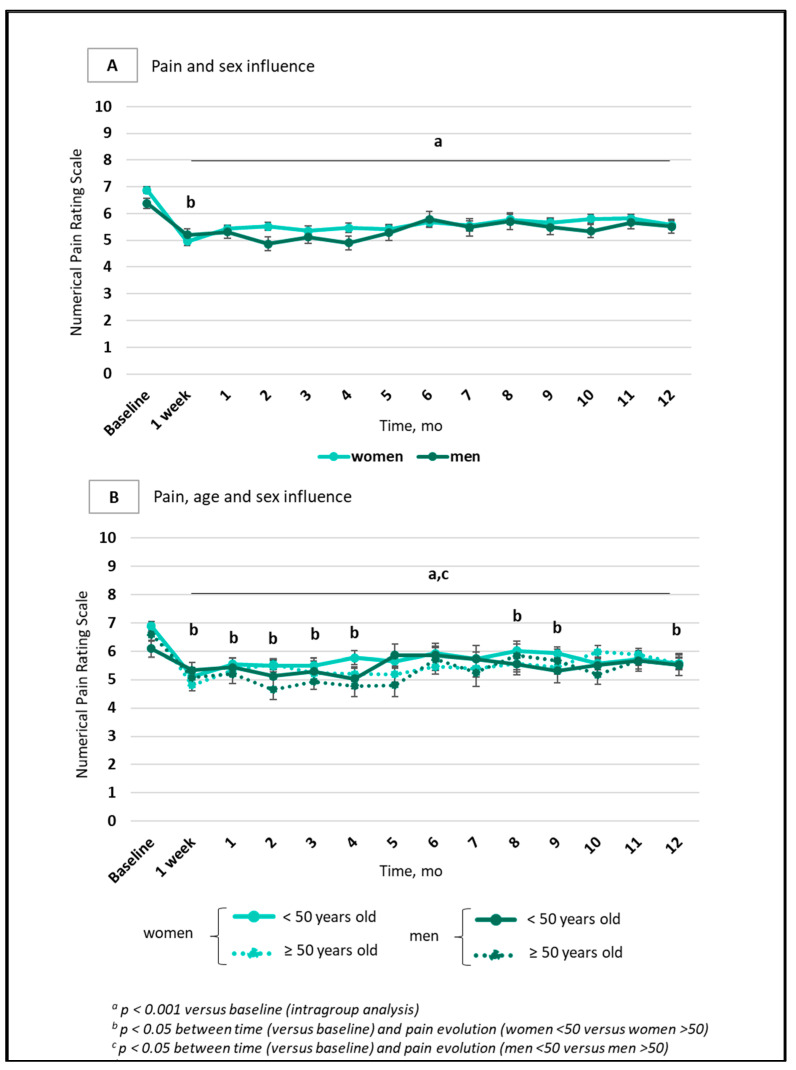
Changes in pain scores according to sex in 256 patients with chronic refractory pain and a single ketamine administration. Data are presented as mean (SEM). (**A**). Pain evolution over one year depending on sex. The light blue line represents women (*n* = 181) and dark green line represents men (*n* = 59). (**B**). Pain evolution over one year depending on sex and age. The light full line represents women < 50 years old (*n* = 86); the light dotted line represents women ≥ 50 years old (*n* = 95). The dark full line represents men < 50 years old (*n* = 31); the dark dotted line represents men ≥ 50 years old (*n* = 28).

The sex x time interaction was significant for differences in the evolution of pain between women and men (*p* = 0.032) (Figure 3A). When pain evolution over one year was analyzed by age (<50 years; ≥50 years), men aged < 50 years had lower pain than those aged ≥ 50 years (*p* = 0.045) (Figure 3B); there were no other differences in variables recorded at baseline (see Supplementary Table S2).

**Figure 4:**
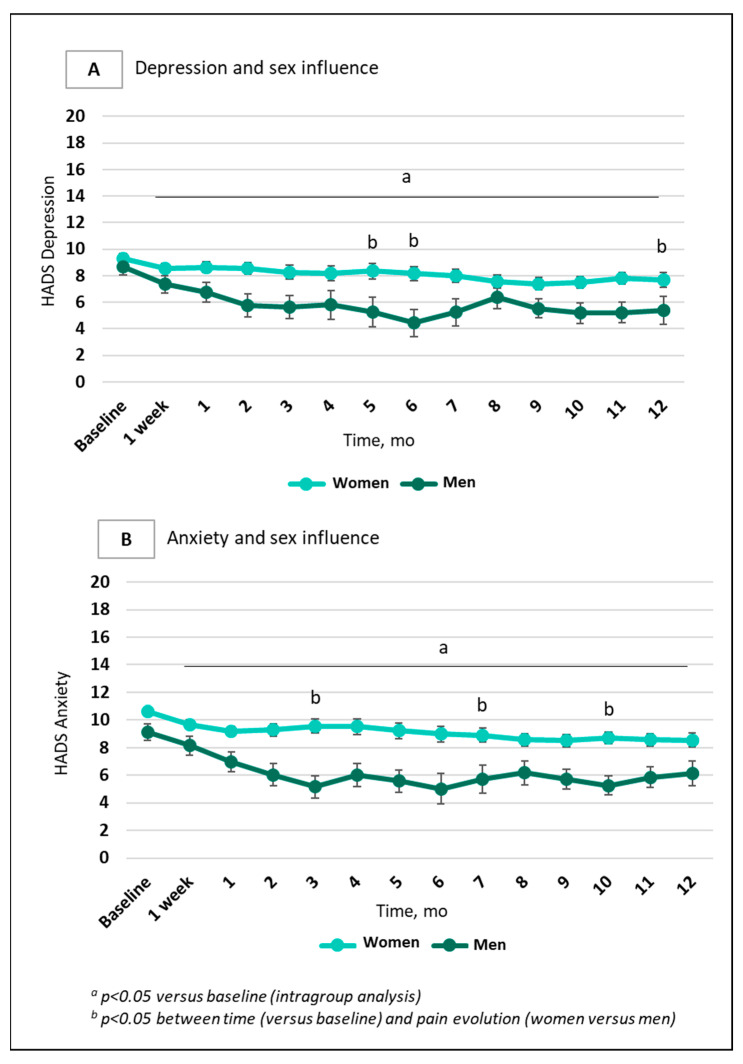
Changes in depression and anxiety scores according to sex in 256 patients with chronic refractory pain and a single ketamine administration. Data are presented as mean (SEM). (**A**). Depression evolution over one year depending on sex. The light blue line represents women (*n* = 180) and dark green line represents men (*n* = 57); (**B**). Anxiety evolution over one year depending on sex. The light blue line represents women (*n* = 180) and dark green line represents men (*n* = 61).

Concerning emotional aspects evaluated using the HAD scale in the total sample, women presented higher depression scores (*p* = 0.004) (Figure 4A) and anxiety scores (*p* = 0.002) (Figure 4B) than men.

As women and men were not comparable in terms of pathology (fibromyalgia: 52.1% of women vs. 21.0% of men, *p* < 0.001), a sensitivity analysis was conducted to match women and men according to pathology and age. The findings for 62 women and 62 men (comparable for fibromyalgia: 21.0% of women vs. 21.0% of men, *p* = 1.00) were analogous concerning the aforementioned results for pain, depression and anxiety evolution.

The mean mental health and mean physical health dimension scores of the SF-12 did not change depending on sex between baseline and month 12.

The cumulative ketamine dose, naïve ketamine profile and route of administration were not different between sexes. Overall, concomitant treatments at baseline were maintained during the year of follow-up, apart from antidepressants and adjuvants (*p* < 0.05) (see Supplementary Figure S4).

## 4. Discussion

In this study, in sample of 585 patients suffering from chronic refractory pain and treated with (R,S)-ketamine showed no differences in pain, anxiety or ketamine dosage -according to sex or age (Figure 2 and Figure 3). Similar results were found in a subgroup of 329 patients who received ketamine administration during the year of follow-up [3], but the 256-patient subgroup did show differences.

In the subgroup of 256 patients who received a single ketamine administration [1], significant differences in pain, depression and anxiety were observed according to sex and age (Figure 4). A significant improvement in men compared to women was observed, especially after 1 week, and a similar finding has been described in the context of depression [6]. This striking effect of a single administration may be linked to the behavioral, molecular and structural effects of ketamine, as described in the literature [24]. The biological mechanisms driving sex-related variations in ketamine’s effects remain incompletely understood, although differences in ketamine metabolism may play a role. For instance, women have been found to exhibit higher concentrations of three ketamine metabolites after administration, two of them (dehydronorketamine; hydroxynorketamine metabolite) showing an inverse correlation with dissociative and psychotomimetic symptoms compared to men [35]. Additionally, preclinical studies have shown differences in the metabolism of N-methyl-D-aspartate receptor antagonists between male and female rats. Specifically, female rats exhibited slower ketamine metabolism after injection compared to male rats [36]. In humans, with one intravenous 0.5 mg/kg ketamine infusion (the most common dosage in our study), the plasma levels of ketamine and norketamine (a powerful analgesic metabolite obtained via N-demethylation) were higher in males than in females, while hydroxynorketamine levels (generated via catalysis by CYP450 isoforms) were not different [37]. As N-demethylase activity is influenced by steroid status and inhibited by progesterone [38], more efficient N-demethylation in men may be one of the metabolic keys to explaining differences in ketamine analgesia. Furthermore, opioid consumption at baseline was higher in men than in women: synergy between ketamine and opioids may indeed also amplify the analgesic effect of ketamine in men. In humans, heterogeneous differences between women and men have been reported in relation to ketamine metabolites, hepatic clearance, biomarkers and side effects [39,40]. Sigtermans and colleagues reported that S-ketamine is metabolized faster metabolism in females, with greater effects observed on cardiac output and heat-induced pain [41].

Sex-specific immunomodulatory and anti-inflammatory actions of ketamine have also been described [21,42], with different trajectories of changes in certain cytokine [42]. The baseline inflammatory status may be more pronounced in men (often with neuropathic pain) than in women (with fibromyalgia), and ketamine may have exerted a strong immediate anti-inflammatory effect with the first dose, with little inflammation left and less pain relief in administrations following. In older persons and ≥50-year-old men, who have an even higher inflammatory status, inflammaging and higher IL6 concentrations, ketamine prevents escape and a drop in analgesia with just one administration.

Furthermore, in this study, we found no differences in adverse effects, but it would be interesting to explore their occurrence more specifically as a function of gender, as shown in a recent study [43]. For example, Morgan et al. demonstrated that intravenous ketamine infusions lead to greater sensitivity to verbal and subjective memory impairments in men compared to women (0.5 and 1.3 mg/kg) [44]. Another study revealed stronger depersonalization and amnestic symptoms after ketamine infusion in young male participants, with the effect potentiated by age, suggesting a sex-specific protective effect of older age [45].

Observational studies, while valuable for observing phenomena in real-life contexts, are subject to a number of biases that can affect the validity of the results. This real-life study has the biases inherent in observational studies, namely, the lack of a control group and missing data. Attrition bias also occurs when participants drop out of a study, introducing a bias in the results. Finally, the diversity of ketamine administration protocols represents a strength of this study, but also a significant bias with regard to the real efficacy of ketamine. Observational research remains valuable in providing the information needed to improve medical decision making.

It is difficult to make specific recommendations given the potential biases in this study. However, the use of ketamine in the management of chronic pain should be tailored to the patient’s age and gender, as these factors may influence its efficacy and potential for adverse events. Older patients may require dose adjustments due to altered metabolism and increased susceptibility to side effects such as cognitive dysfunction. Women may metabolize S-ketamine more rapidly and may require different dosing strategies than men, as they may experience greater effects on pain perception. Clinicians should monitor these variables closely to optimize outcomes and minimize risks.

In conclusion, with repeated ketamine administrations in chronic pain patients, there are no sex and age differences in ketamine efficacy or safety, pain, anxiety or depression. Future trials should, however, focus on sex and age differences with a single ketamine administration, especially in older persons, as it may be more beneficial for men than women, especially after one week. Similar findings for refractory pain and depression, a frequent comorbidity of pain, suggest a common template for ketamine’s mode of action and warrant further studies of ketamine use in patients with refractory chronic pain.

## Figures and Tables

**Table 1:** Demographics and clinical characteristics of patients at baseline before ketamine administration. Data are presented as number of patients (percentages), mean ± standard deviation or median [25th; 75th percentiles]. In this table, missing data are not imputed. DN4, Douleur Neuropathique 4; HADS, Hospital Anxiety and Depression Scale; IV, intravenous; NSAIDs, Non-Steroidal Anti-Inflammatory Drugs; SF-12, 12-item Short Form Health Survey; WHO, World Health Organization; NS: not significant. ^a^ DN4: except for fibromyalgia. ^b^ Step 2 (WHO) opioids: dihydrocodeine; ibuprofene codeine; paracetamol codeine; paracetamol opium; paracetamol opium caffeine; paracetamol tramadol; tramadol; tramadol deketopofene. ^c^ Step 3 (WHO) opioids: morphine; oxycodone; fentanyl; buprenorphine.

	Total (*n* = 585)	Women (*n* = 443)	Men (*n* = 142)	*p*-Values
**Demographics**				
Age (years)	51.1 ± 11.2	51.3 ± 11.1	50.5 ± 11.6	NS
**Pain-Related**				
Pain etiology				
Fibromyalgia	287 (49.1)	251 (56.7)	36 (25.4)	<0.001
Peripheral neuropathic pain	173 (29.6)	107 (24.2)	66 (46.5)	<0.001
DN4 ^a^ (0–10) (*n* = 260/168/92)	5.4 ± 2.2	5.5 ± 2.3	5.3 ± 2.2	NS
DN4 ^a^ ≥4	217 (83.6)	141 (83.9)	76 (82.6)	NS
Average pain intensity (0–10) (*n* = 555/422/133)	6.8 ± 1.8	6.9 ± 1.8	6.4 ± 1.7	0.0051
<3	8 (1.4)	6 (1.4)	2 (1.5)	
3 to 6	217 (39.2)	154 (36.5)	63 (47.4)	0.065
≥7	330 (59.7)	262 (62.1)	68 (51.1)	
Number of pain paroxysms (*n* = 347/258/89)	4 [2.5; 8]	4 [2; 8]	4 [3; 7]	NS
Maximal pain intensity (0–10) (*n* = 557/423/134)	8.3 ± 1.5	8.3 ± 1.6	8.3 ± 1.4	NS
<3	4 (0.7)	4 (1.0)	0 (0)	
3 to 6	51 (9.2)	37 (8.8)	14 (10.5)	NS
≥7	502 (90.1)	382 (90.3)	120 (89.6)	
**Ketamine**				
Ketamine naive	224 (38.3)	162 (36.6)	62 (43.7)	NS
IV route	506 (86.5)	379 (85.5)	127 (89.4)	NS
IV cumulative dose (mg) (*n* = 506/379/127)	391.5 ± 320.4	395.1 ± 331.7	380.6 ± 284.8	NS
**Emotional Aspects**				
HADS. anxiety score (0–21) (*n* = 554/416/138)	10.4 ± 4.4	10.8 ± 4.3	8.9 ± 4.3	<0.001
≤7	164 (29.6)	106 (25.5)	58 (42.0)	
8 to 10	122 (22.0)	91 (21.9)	31 (22.5)	<0.001
≥11	268 (48.4)	219 (52.6)	49 (35.5)	
HADS. depression score (0–21) (*n* = 549/416/133)	9.0 ± 4.3	9.1 ± 4.4	8.7 ± 4.2	NS
≤7	213 (38.8)	161 (38.7)	52 (39.1)	
8 to 10	128 (23.3)	95 (22.8)	33 (24.8)	NS
≥11	208 (37.9)	160 (38.5)	48 (36.1)	
**Quality of Life**				
SF-12. physical score (*n* = 513/388/125)	29.1 ± 8.1	28.9 ± 7.9	29.8 ± 8.6	NS
SF-12. mental score (*n* = 513/388/125)	39.0 ± 10.8	38.7 ± 10.5	40.1 ± 11.5	NS
**Concomitant Drugs**				
Number of treatments	3.3 ± 1.9	3.3 ± 1.8	3.4 ± 2.1	NS
Paracetamol/NSAIDs	244 (42.5)	187 (42.8)	57 (41.6)	NS
Step 2 opioids ^b^. nefopam	289 (50.3)	226 (51.7)	63 (46.0)	NS
Step 3 opioids ^c^	87 (15.2)	56 (12.8)	31 (22.6)	0.005
Antidepressants	373 (65.0)	296 (67.7)	77 (56.2)	0.014
Antiepileptics	213 (37.1)	153 (35.0)	60 (43.8)	NS
Adjuvants	117 (20.4)	86 (19.7)	31 (22.6)	NS
Hypnotics/sedatives	97 (16.9)	67 (15.3)	30 (21.9)	NS
Anxiolytics	165 (28.7)	132 (30.2)	33 (24.1)	NS
Antipsychotics	33 (5.7)	26 (6.0)	7 (5.1)	NS
Others	47 (8.2)	37 (8.5)	10 (7.3)	NS
None	32 (5.6)	22 (5.0)	10 (7.3)	NS

## Data Availability

The datasets generated and/or analyzed during the current study are available from the corresponding author upon reasonable request.

## Supplementary Materials

The following supporting information can be downloaded at https://www.mdpi.com/article/10.3390/jcm14124269/s1, Figure S1: Depression and anxiety evolution according to sex in 585 patients with chronic refractory pain; Figure S2: Pain evolution according to sex and age in 329 patients with chronic refractory pain and repeated ketamine administration; Figure S3: Depression and anxiety evolution according to sex in 329 patients with chronic refractory pain and repeated ketamine administration; Figure S4: Pharmacological treatments during the year of follow-up in 256 patients with chronic refractory pain and a single ketamine administration; Table S1: Demographics and clinical characteristics at baseline before ketamine administration between women and men in cohort with single ketamine administration; Table S2: Demographics and clinical characteristics at baseline before ketamine administration between young and older men in cohort with single ketamine administration.
