# Supplementary material for: Sex and Age Differences in Ketamine Efficacy and Safety in Chronic Pain Alleviation
Source: J Clin Med. 2025 Jun 16;14(12):4269. doi: 10.3390/jcm14124269 (PMC12194336; doi:10.3390/jcm14124269)
Supplement: Supplementary file 1 [file jcm-14-04269-s001.zip › jcm-3584458-supplementary.pdf]

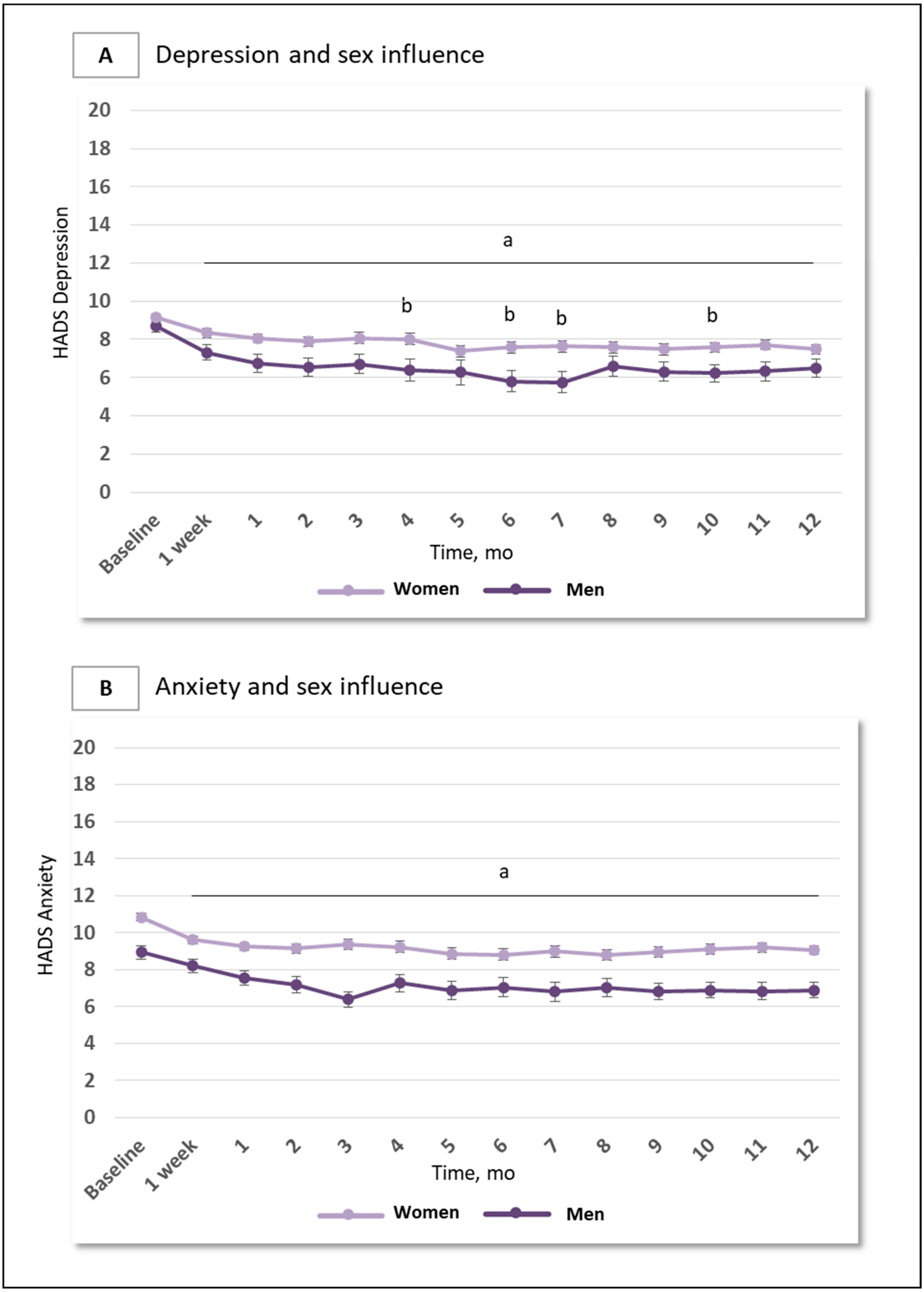

Figure S1 │ Depression and anxiety evolution according to sex in 585 patients with chronic refractory pain.

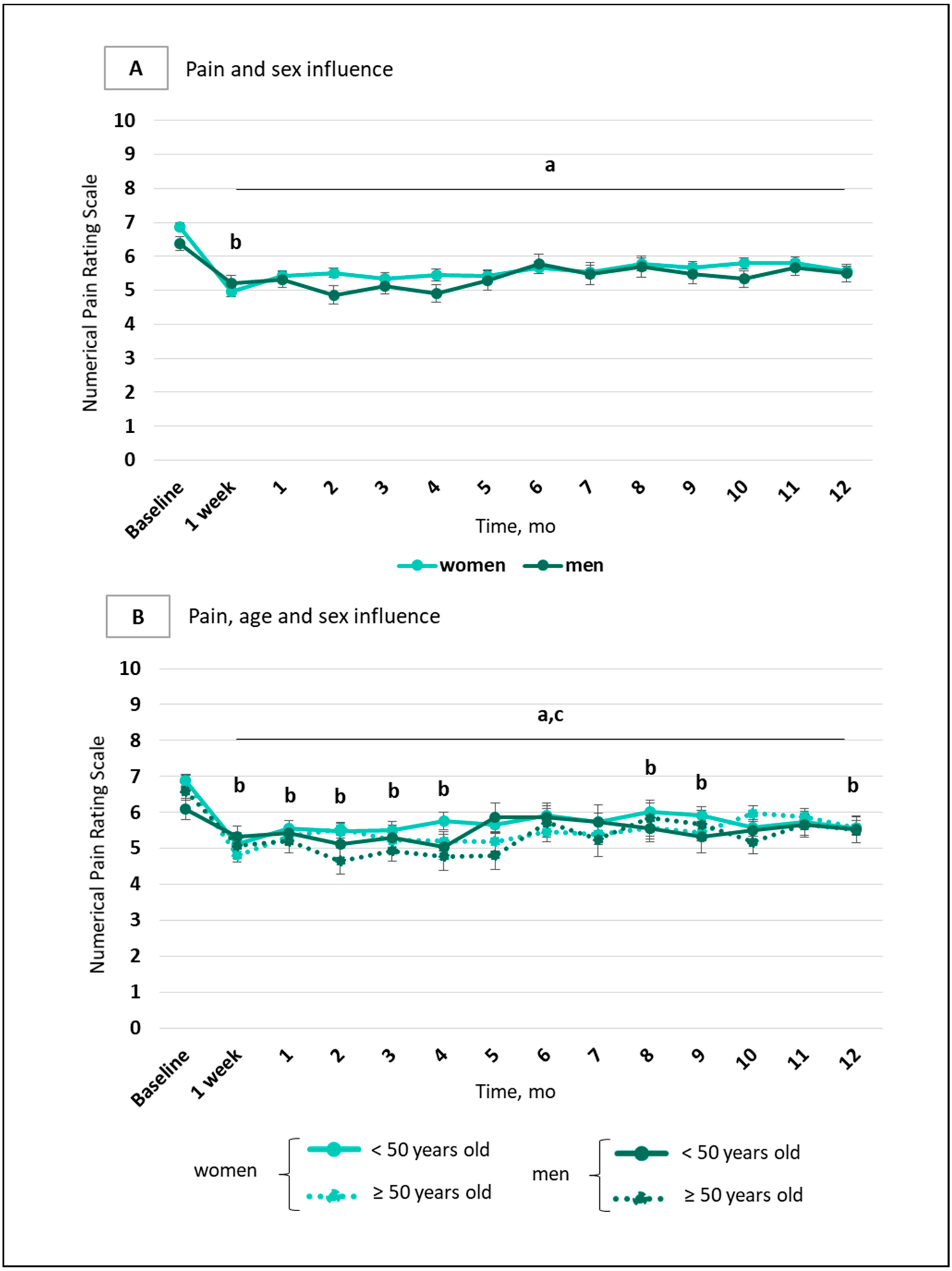

Figure S2 │ Pain evolution according to sex and age in 329 patients with chronic refractory pain and repeated ketamine administration.

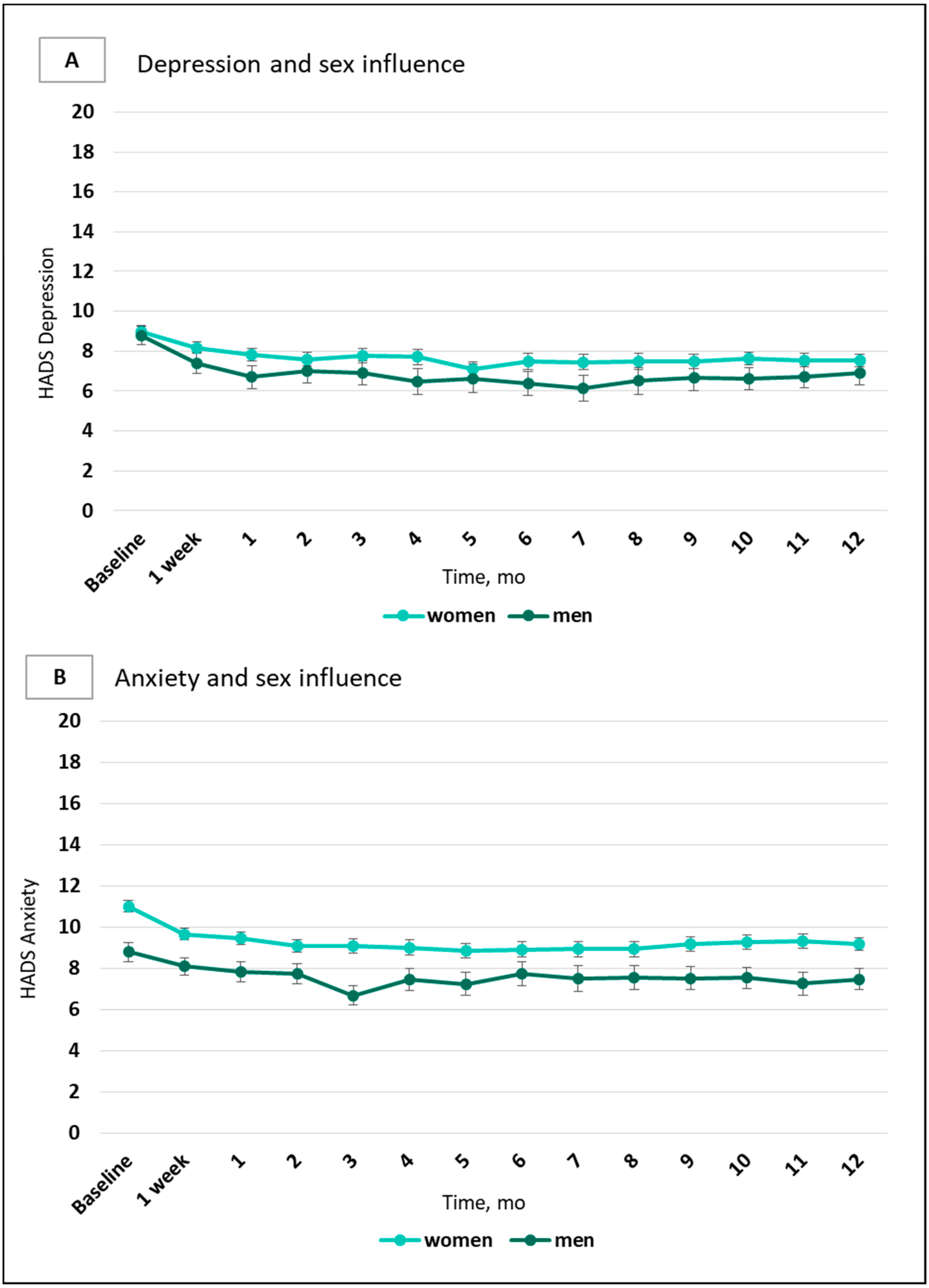

Figure S3 | Depression and anxiety evolution according to sex in 329 patients with chronic refractory pain and repeated ketamine administration.

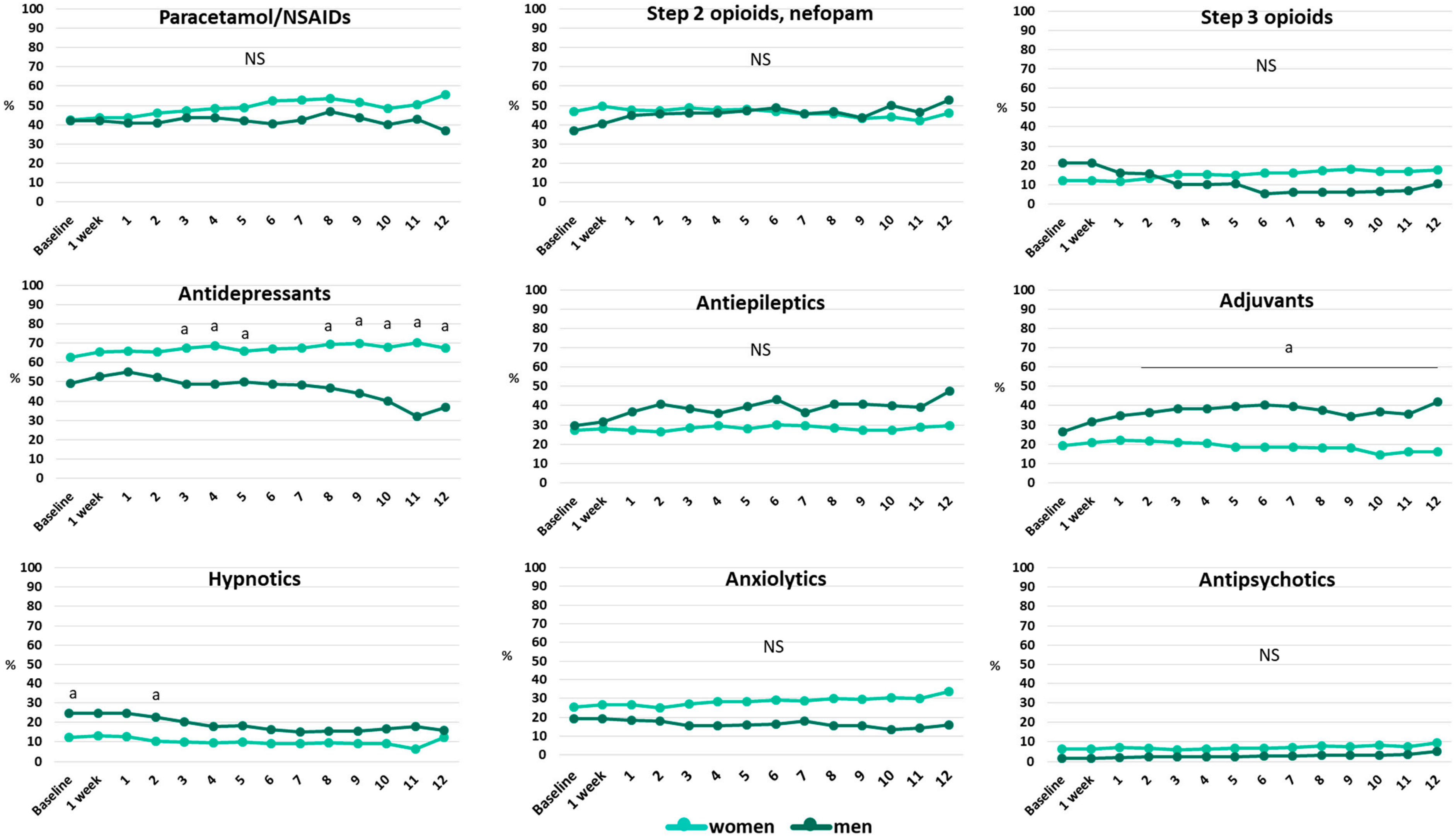

Fig S4│ Pharmacological treatments during the year of follow-up in 256 patients with chronic refractory pain and a single ketamine administration.

Table S1. Demographics and clinical characteristics at baseline before ketamine administration between women and men in cohort with single ketamine administration. Data are presented as number of patients (percentages). mean ± standard deviation. or median [25th; 75th percentiles]. In this table, missing data are not imputed. DN4. Douleur Neuropathique 4; HADS. Hospital Anxiety and Depression Scale; IV. intravenous; NSAIDs. Non-Steroidal Anti-Inflammatory Drugs; SF-12. 12-item Short Form health survey; WHO. World Health Organization. [a] DN4: except for fibromyalgia. [b] Step 2 (WHO) opioids: dihydrocodeine; ibuprofene-codeine; paracetamol-codeine; paracetamol-opium; paracetamol-opium-cafeine; paracetamol-tramadol; tramadol; tramadol-deketopofene. [c] Step 3 (WHO) opioids: morphine; oxycodone; fentanyl; buprenorphine.

**Characteristics**	**Total (n=256)**	**WOMEN (n=194)**	**MEN (n=62)**	**p-values**
Age, mean (SD), y	50.7 (11.5)	51.2 (11.4)	49.3 (11.7)	0.273
**Pain-related**				
Pain etiology				
Fibromyalgia	36 (25.4)	101 (52.1)	13 (21.0)	<0.001
Peripheral neuropathic pain	72 (28.1)	45 (23.2)	27 (43.6)	0.003
DN4 (n = 92)				
Mean (SD) score	5.2 (2.3)	5.4 (2.2)	5.0 (2.4)	0.386
≥4, No./total No. (%)	76/92 (82.6)	68/82 (82.9)	31/43 (72.1)	0.170
Mean pain intensity				
Mean (SD) score	6.8 (1.8)	6.9 (1.7)	6.4 (1.9)	0.073
No./total No. (%) of patients				
<3	6/240 (2.5)	5/181 (2.8)	1/59 (1.7)	
3 to 6	91/240 (37.9)	61/181 (33.7)	30/59 (50.9)	0.057
≥7	143/240 (59.6)	115/181 (63.5)	28/59 (47.5)	
No. of pain paroxysms, median (IQR) *(n=244/185/59)*	4 (2.5-7)	4 (2.5-7)	4 (2.5-5)	0.982
Maximal pain intensity				
Mean (SD) score	8.2 (1.6)	8.2 (1.6)	8.2 (1.4)	0.949
No./total No. (%) of patients				
<3	3/244 (1.2)	3/185 (1.6)	0/59	0.915

Characteristics	Total (n=256)	WOMEN (n=194)	MEN (n=62)	p-values
3 to 6	23/244 (9.4)	17/185 (9.2)	6/59 (10.2)	
≥7	218/244 (89.3)	165/185 (89.2)	53/59 (89.8)	
**Ketamine**				
Ketamine naive	126 (49.2)	89 (45.9)	37 (59.7)	
IV route	227 (88.7)	172 (88.7)	55 (88.7)	NS
IV cumulative dose	207.2 (115.3)	209.8 (114.6)	199.3 (118.4)	
**Emotional aspects**				
HADS anxiety score				
Mean (SD) score	10.2 (4.4)	10.6 (4.3)	9.1 (4.7)	0.020
No./total No. (%) of patients				
≤7	73/241 (30.3)	46/180 (25.6)	27/61 (44.3)	
8 to 10	57/241 (23.7)	47/180 (26.1)	10/61 (16.4)	0.023
≥11	111/241 (46.1)	87/180 (48.3)	24/61 (39.3)	
HADS depression score				
Mean (SD) score	9.2 (4.5)	9.3 (4.5)	8.6 (4.5)	0.330
No./total No. (%) of patients				
≤7	95/237 (40.1)	73/180 (40.6)	22/57 (38.6)	
8 to 10	53/237 (22.4)	39/180 (21.7)	14/57 (24.6)	0.906
≥11	89/237 (37.6)	68/180 (37.8)	21/57 (36.8)	
**Quality of life**				
SF-12. physical score, mean (SD) *(n=219/165/54)*	30.1 (8.1)	29.5 (7.8)	31.8 (9.0)	0.08
SF-12. mental score, mean (SD) *(n=219/165/54)*	38.5 (10.7)	37.8 (10.5)	40.9 (11.3)	0.066
**Concomitant drugs**				
Number of treatments	3.0 (1.8)	3.0 (1.8)	2.9 (1.7)	0.645
Paracetamol/NSAIDs	104 (42.5)	80 (42.6)	24 (42.1)	1.0
Step 2 opioids[b]. nefopam	109 (44.5)	88 (46.8)	21 (36.8)	0.224
Step 3 opioids[c]	35 (14.3)	23 (12.2)	12 (21.1)	0.128
Antidepressants	146 (59.6)	118 (62.8)	28 (49.1)	0.090
Antiepileptics	68 (27.8)	51 (27.1)	17 (29.8)	0.736
Adjuvants	51 (20.8)	36 (19.2)	15 (26.3)	0.266
Hypnotics/sedatives	37 (15.1)	23 (12.2)	14 (24.6)	0.033
Anxiolytics	59 (24.1)	48 (25.5)	11 (19.3)	0.381
Antipsychotics	13 (5.3)	12 (6.4)	1 (1.8)	0.309
Others	16 (6.5)	14 (7.4)	2 (3.5)	0.375
None	19 (7.8)	13 (6.9)	6 (10.5)	0.399

Table S2. Demographics and clinical characteristics at baseline before ketamine administration between young men and old men in cohort with single ketamine administration. Data are presented as number of patients (percentages). mean ± standard deviation. or median [25th; 75th percentiles]. In this table, missing data are not imputed. DN4. Douleur Neuropathique 4; HADS. Hospital Anxiety and Depression Scale; IV. intravenous; NSAIDs. Non-Steroidal Anti-Inflammatory Drugs; SF-12. 12-item Short Form health survey; WHO. World Health Organization. [a] DN4: except for fibromyalgia. [b] Step 2 (WHO) opioids: dihydrocodeine; ibuprofene-codeine; paracetamol-codeine; paracetamol-opium; paracetamol-opium-cafeine; paracetamol-tramadol; tramadol; tramadol-deketopofene. [c] Step 3 (WHO) opioids: morphine; oxycodone; fentanyl; buprenorphine.

**Characteristics**	**Total MEN (n=62)**	**MEN<50 (n=34)**	**MEN≥50 (n=28)**	**p-values**
Age, mean (SD), y	49.3 (11.7)	41.1 (6.9)	59.3 (7.8)	<0.001
**Pain-related**				
Pain etiology				
Fibromyalgia	13 (21.0)	9 (26.5)	4 (14.3)	0.241
Peripheral neuropathic pain	27 (43.6)	15 (44.1)	12 (42.9)	0.921
DN4 (n = 92)				
Mean (SD) score	5.0 (2.4)	4.6 (2.5)	5.4 (2.3)	0.289
≥4, No./total No. (%)	31/43 (72.1)	15/23 (65.2)	16/20 (80.0)	0.281
Mean pain intensity				
Mean (SD) score	6.4 (1.9)	6.3 (1.8)	6.5 (1.9)	0.718
No./total No. (%) of patients				
<3	1/59 (1.7)	1/31 (3.2)	0/28	
3 to 6	30/59 (50.9)	15/31 (48.4)	15/28 (53.6)	0.609
≥7	28/59 (47.5)	15/31 (48.4)	13/28 (46.4)	
No. of pain paroxysms, median (IQR) *(n=33/19/14)*	4 (2.5-5)	4 (2.5-5)	4 (2-4)	0.630
Maximal pain intensity				
Mean (SD) score	8.2 (1.4)	8.3 (1.3)	8.0 (1.5)	0.454
No./total No. (%) of patients				
<3	0/59	0/32 (1.6)	0/27	0.278
3 to 6	6/59 (10.2)	2/32 (6.3)	4/27 (14.8)	

Characteristics	Total MEN (n=62)	MEN<50 (n=34)	MEN≥50 (n=28)	p-values
≥7	53/59 (89.8)	30/32 (93.8)	23/27 (85.2)	
**Ketamine**				
IV cumulative dose	199.3 (118.4)	193.5 (92.6)	206.2 (145.2)	NS
**Emotional aspects**				
HADS anxiety score				
Mean (SD) score	9.1 (4.7)	8.6 (4.5)	9.7 (4.9)	0.373
No./total No. (%) of patients				
≤7	27/61 (44.3)	16/33 (48.5)	11/28 (39.3)	0.580
8 to 10	10/61 (16.4)	4/33 (12.1)	6/28 (21.4)	
≥11	24/61 (39.3)	13/33 (39.4)	11/28 (39.4)	
HADS depression score				
Mean (SD) score	8.6 (4.5)	8.8 (4.9)	8.5 (4.0)	0.804
No./total No. (%) of patients				
≤7	22/57 (38.6)	12/32 (37.5)	10/25 (40.0)	0.771
8 to 10	14/57 (24.6)	7/32 (21.9)	7/25 (28.0)	
≥11	21/57 (36.8)	13/32 (40.6)	8/25 (32.0)	
**Quality of life**				
SF-12. physical score, mean (SD) *(n=219/165/54)*	31.8 (9.0)	32.5 (8.6)	30.9 (9.4)	0.499
SF-12. mental score, mean (SD) *(n=219/165/54)*	40.9 (11.3)	40.7 (12.5)	41.0 (9.9)	0.927
**Concomitant drugs**				
Number of treatments	2.8 (1.7)	2.9 (2.0)	2.9 (1.5)	0.962
Paracetamol/NSAIDs	24 (42.1)	9 (30.0)	15 (55.6)	0.051
Step 2 opioids[b]. nefopam	21 (36.8)	12 (40.0)	9 (33.3)	0.602
Step 3 opioids[c]	12 (21.1)	7 (23.3)	5 (18.5)	0.656
Antidepressants	28 (49.1)	15 (50.0)	13 (48.2)	0.889
Antiepileptics	17 (29.8)	7 (23.3)	10 (37.0)	0.259
Adjuvants	15 (26.3)	7 (23.3)	8 (29.6)	0.590
Hypnotics/sedatives	14 (24.6)	10 (33.3)	4 (14.8)	0.105
Anxiolytics	11 (19.3)	7 (23.3)	4 (14.8)	0.416
Antipsychotics	1 (1.8)	1 (3.3)	0 (0)	0.339
Others	2 (3.5)	2 (6.7)	0 (0)	0.172
None	6 (10.5)	5 (16.7)	1 (3.7)	0.111
